# Hashtags as signals of political identity: #BlackLivesMatter and #AllLivesMatter

**DOI:** 10.1371/journal.pone.0286524

**Published:** 2023-06-08

**Authors:** Maia Powell, Arnold D. Kim, Paul E. Smaldino

**Affiliations:** 1 Department of Applied Mathematics, University of California, Merced, CA, United States of America; 2 Department of Cognitive & Information Sciences, University of California, Merced, CA, United States of America; 3 Santa Fe Institute, Santa Fe, NM, United States of America; Carnegie Mellon Univeristy, UNITED STATES

## Abstract

We investigate perceptions of tweets marked with the #BlackLivesMatter and #AllLivesMatter hashtags, as well as how the presence or absence of those hashtags changed the meaning and subsequent interpretation of tweets in U.S. participants. We found a strong effect of partisanship on perceptions of the tweets, such that participants on the political left were more likely to view #AllLivesMatter tweets as racist and offensive, while participants on the political right were more likely to view #BlackLivesMatter tweets as racist and offensive. Moreover, we found that political identity explained evaluation results far better than other measured demographics. Additionally, to assess the influence of hashtags themselves, we removed them from tweets in which they originally appeared and added them to selected neutral tweets. Our results have implications for our understanding of how social identity, and particularly political identity, shapes how individuals perceive and engage with the world.

## Introduction

Individuals regularly broadcast information about who they are in public forums, and it is widely acknowledged by social scientists that an important function of public communication is to signal one’s real or potential membership in some categorizable subset of individuals [1–5]. Identity signaling serves numerous social functions, such as indicating one’s commitment to particular groups [6–8] and facilitating cooperative assortment for activities requiring cooperation or coordination [5, 9–11]. Assortative signaling can be overt, so that information is widely received by diverse audiences, or covert, where information is encrypted so that only audiences “in the know” reliably perceive the identity-related content [12, 13]. Covert signals can be beneficial for the transmitter because they allow for individuals to strategically alter the clarity of their messages, imbuing them with cryptic or indirect meanings when they are likely to be viewed by hostile audiences [14]. Despite its use in facilitating cooperation between similar individuals, however, strategic identity signaling is not always aligned with societal good. For example, white supremacists have likely used covert signals on online social networks such as Twitter to coordinate with others while avoiding widespread detection [15].

Social identity provides a lens that shapes and alters how humans perceive the world. Pertinently, a more general phenomenon exists of cultural influences on cognitive development [16, 17]. In the contemporary United States, political partisanship has become one of the most salient identity categories, correlating with variation on traits from religiosity to gun ownership to television show preference [18–20]. Accordingly, Americans on the political left and right appear to inhabit very different mental worlds. Differences in psychological traits, including need for cognition, tolerance for ambiguity, and need to evaluate, have been found to correlate with differences in political ideology [21]. Further, left-right political orientation appears to correlate with reliably different personality profiles, resulting in correspondingly different behavioral patterns [22]. The phenomenon of affective polarization is at this point well described, whereby political decisions of left and right partisans are driven more by opposition to the other side than by any positive policy preferences [23–25]. Moreover, identical stimuli can be perceived in a dramatically different light by left and right partisans [26]. For example, Kahan *et al.* [27] presented participants with identical footage of a protest and asked about their support for police intervention to quell it. Republican participants were more likely than Democrats participants to support police action when told the protest was in opposition to the military’s policy of “don’t ask, don’t tell” outside a military recruitment office, while the effect was reversed when participants were told that the protesters were opposing abortion outside an abortion clinic.

In the digital age, social media platforms such as Twitter have become wide forums for partisan identity signaling. A particularly interesting affordance for identity signaling on social media is the hashtag. First introduced on Twitter in 2007 as a way to categorize messages for more refined searching [28], the function of hashtags has since evolved. Hashtags can serve as nuanced communicative tags, marking tweets with contextual information that highlights or excludes potential implicatures for different audiences. Tagging tweets with hashtags connected with social movements, such as #MeToo or #BlackLivesMatter, indicates to audiences that the message in the tweet is directly connected with those movements. This can have implications for how the message is perceived. A recent study by Rho and Mazmanian [29] found that the presence of a hashtag in a tweet sharing new stories led to those stories being perceived as more partisan. It seems possible that hashtags can function as identity signals, marking a tweet—and by extension the tweet’s author—as belonging or declaring allegiance to particular identity groups.

Among the most widespread and influential socio-political hashtags that have emerged in recent years is #BlackLivesMatter, which gained significance after the murders of Trayvon Martin and Michael Brown and subsequent lack of criminal convictions for their killers in 2013 and 2015, respectively [30, 31]. The hashtag later evolved to bring awareness to many other acts of injustice against Black members of the population, primarily by police. In response, the hashtag #AllLivesMatter was created to assert “colorblind” attitudes ostensibly at odds with sentiments expressed by #BlackLivesMatter [32–35]. Although neither hashtag is formally associated with any political party, they have over time become entangled in the increasingly polarized landscape of American political identity [35, 36]. Recent studies found that Democrats show increased support for the Black Lives Matter movement compared with Republicans [37, 38], though neither study looked specifically at hashtags. Less evidence exists about partisanship and the All Lives Matter movement, though a recent qualitative analysis argued that the movement has been far more often invoked by Republican political candidates than by Democrats [39]. Given the extent of polarization in the U.S. around political identities, it seems possible not only that perceptions of the two hashtags may differ wildly between left and right partisans, but even that the hashtags themselves may serve as a sort of identity signal, providing reliable context cues regarding how the author of an online message wishes their statement to be interpreted.

In this paper we report on our investigations into how political identity moderates the perception of tweets tagged with the #BlackLivesMatter and #AllLivesMatter hashtags, expecting that partisans on the left would view the former more favorably than the latter, with the reverse effect for partisans on the political right. We were particularly interested in participants’ perceptions of the tweets as offensive or racist. Moreover, we investigated the specific information content of the hashtags themselves in fueling partisan perceptions. We did this by artificially removing the hashtags from tweets in which they initially appeared, as well as by appending them to tweets completely unconnected to either movements. We investigated a number of possible predictors of affective responses to tweets, with a particular emphasis on political identity—an emphasis that, as we shall see, appears to have been warranted.

We found that perceptions of tweets marked with the #AllLivesMatter and #BlackLivesMatter hashtags were strongly correlated to political orientation, such that individuals on the political left rate #AllLivesMatter tweets as being more offensive and racist than #BlackLivesMatter tweets, with the reverse effect for #AllLivesMatter tweets. These correlations were moderated by the presence of the hashtags themselves, such that the mere presence of the hashtag tended to strengthen the correlations between ratings and political orientation. Our study indicates that hashtags serve an important role in providing context for the interpretation a tweet’s contents. We further support this assertion by showing that the addition of #AllLivesMatter and #BlackLivesMatter hashtags to otherwise neutral, non-political tweets dramatically increased perceptions that the tweets were both offensive and racist among partisans opposed to respective movements.

## Methods

### Dataset

To obtain a dataset of #AllLivesMatter and #BlackLivesMatter tweets, we used a web crawler [40], which obtains only publicly available tweets via Twitter Advanced Search in compliance with Twitter’s rules (https://help.twitter.com/en/rules-and-policies/twitter-search-policies). We focused on tweets published in the year 2020 in order to constrain the contextual meaning of the tweets to be maximally salient to our participants, who evaluated the tweets in early 2021. That is, we scraped tweets containing either hashtag (“#AllLivesMatter” or “#BlackLivesMatter”, case insensitive), and published between January and December 2020. This resulted in a total of 24 queries (one for each month for each hashtag) and yielded a total of 3,515,489 tweets (2,963,778 #BlackLivesMatter tweets and 551,711 #AllLivesMatter tweets). We then filtered these to create a set of tweets that contained only one hashtag, and had no mention of other Twitter handles and no attachments (pictures, videos, links, etc.). We further filtered the set of tweets manually, so that all tweets placed the hashtag at the very end of the tweet and did not use the hashtag itself as the subject of the tweet’s message (*e.g.*, “My least favorite hashtag is #BlackLivesMatter”). In other words, our interest was in tweets that used the hashtags only as concluding tags.

Neutral tweets were sampled from previous studies in which tweets were evaluated via crowdsourcing and rated as being racist, sexist, both, or neither [41, 42]. We selected tweets from these datasets that were not rated by any participant as either racist or sexist and that appeared to us to be about politically neutral content. Some examples of the topics addressed in these tweets include the weather, food, and traffic.

**Fig 1:**
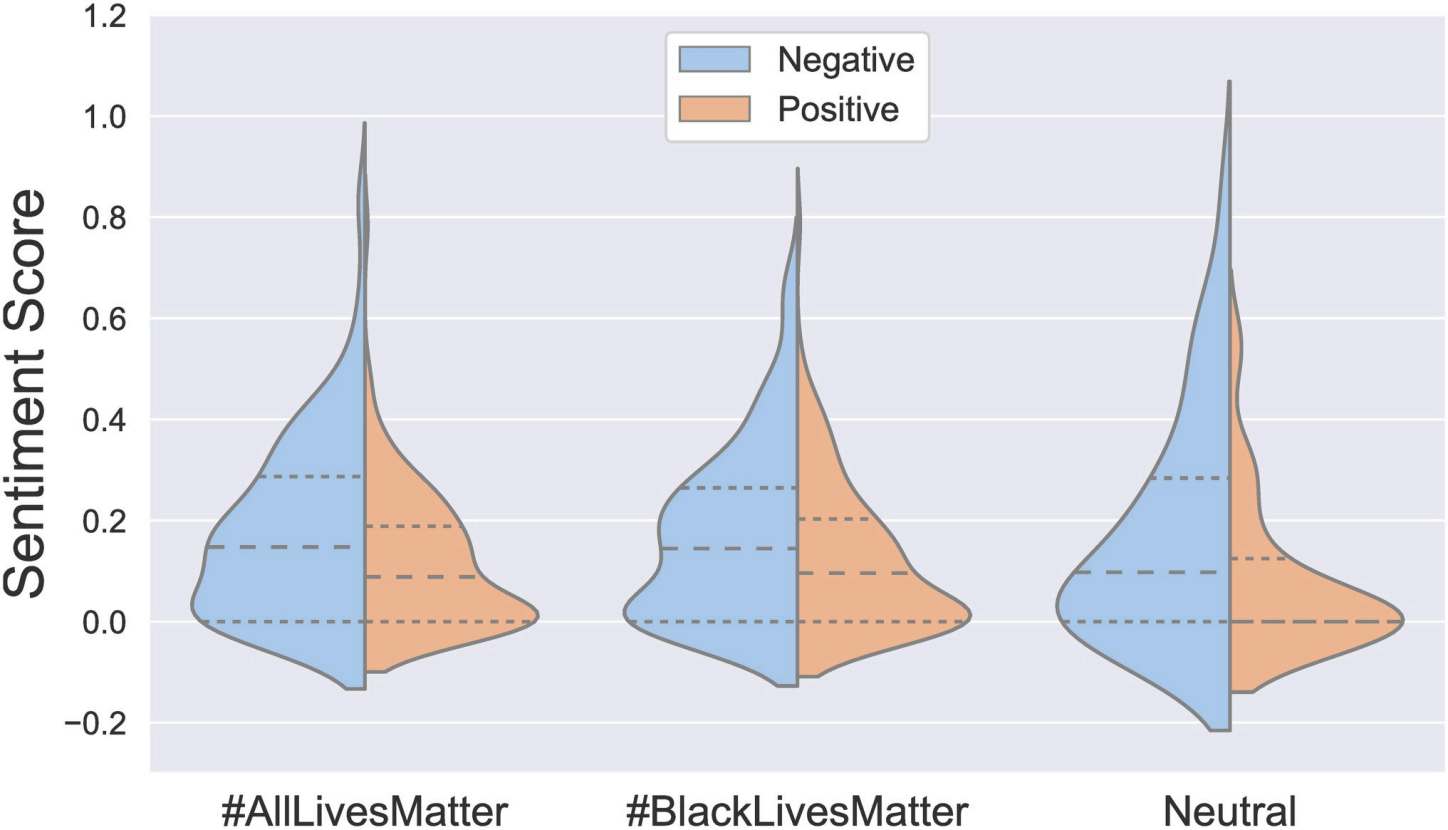
Tweet sentiment scores. Violin plots of positive and negative sentiment scores for #AllLivesMatter, #BlackLivesMatter, and neutral tweets used for this study. Dashed lines represent the means and dotted lines delineate the upper and lower quartiles of each distribution.

We applied a sentiment analysis to the three groups of tweets (#AllLivesMatter tweets, #BlackLivesMatter tweets, and the set of neutral tweets), from the nltk package on Python, which utilizes vader to employ a word-lookup based scoring [43]. The results of that analysis are shown in Fig 1. We observe that all sets of tweets are generally more negative than positive in sentiment. Additionally, we observe minimal differences between sentiment distributions of #AllLivesMatter and #BlackLivesMatter tweets, diminishing the possibility that any differences in the interpretation of these tweets is due to differences in their overall sentiment. The distributions of positive and negative sentiment scores for #AllLivesMatter and #BlackLivesMatter tweets were more similar to one another than either were to the neutral tweets, which perhaps unsurprisingly tended to express substantially weaker sentiments overall.

Each set of tweets was further reduced to a small sample for use in participant surveys, for which we used 300 tweets in total. These were partitioned into ten distinct sets comprised of 30 tweets each. Each set contains 13 #AllLivesMatter tweets, 13 #BlackLivesMatter tweets, and four neutral tweets. The size of these sets was based on the number of tweets our pilot study determined could be reasonably rated by participants without fatigue or attrition, in order for each tweet to be rated by multiple participants. Each participant was randomly assigned one of the ten distinct sets of tweets to evaluate, either with or without hashtags present.

### Survey setup

At the beginning of the survey, each individual was asked to submit written consent to participate in the study. Individuals were prompted to select either “I consent to participate in this study” or “I do not wish to participate in this study” after being shown descriptions of the study’s purpose, procedures, compensation, risks, benefits, and confidentiality. They were also given the right to refuse or withdraw from the study. Following the consent portion, users were then prompted to complete a CAPTCHA verification. If the individual denied consent, the survey ended immediately. If the individual agreed and successfully completed verification, they were next provided with detailed instructions on how to complete the study, as well a necessary definitions. Participants were then presented with 30 tweets in random order and asked to evaluate them on several criteria. For each tweet, participants were instructed to evaluate whether its contents could be perceived as racist, offensive, both or neither, and whether these perceptions applied to (i) themselves personally, (ii) individuals within their social network, and (iii) individuals outside of their social network. The terms “personally”, “within social network”, and “outside of social network” were defined in the instructions, provided in S1 Fig. Our goal in asking participants to imagine how other people were likely to perceive the tweets was to enable us to examine the extent to which participants viewed their own valuations as being related to their social identities rather than as either solely personal views or human universals.

Participants were randomly assigned one of the ten datasets. To document the effect of hashtags on perceptions, some participants were presented tweets with hashtags and the others tweets without hashtags. If a participant was assigned the dataset with hashtags present, they were shown the raw tweets with hashtags already present and neutral tweets with “#AllLivesMatter” or “#BlackLiveMatter” appended. If a participant was assigned the dataset without hashtags present, they were shown the #AllLivesMatter and #BlackLivesMatter tweets with the hashtag omitted and unaltered neutral tweets.

After completion of tweet evaluations, participants were asked to fill out a demographic survey. Individuals were asked about their age, gender, familiarity with hashtags, news consumption, religiosity, and political orientation. We intentionally place the demographic survey *after* the tweet evaluations to ensure participants were not primed to give “identity-typical” responses.

In the United States especially, religiosity tends to have significant, yet complex, effect on an individual’s political views and general identity [44–46]. To gauge religiosity in a more fine-grained way, we utilized a subset of the Centrality of Religiosity Scale (CRS) [47], a measure of the importance of religion in a person’s life. In order to focus on identity-relevant aspects, we selected questions that gauged participation in religious services and membership in religious communities and omitted questions about self evaluations of spirituality. To measure political orientation, we adapted an 11-question survey from the Pew Research Center [48]. Participants were shown a series of two opposing opinions (one “Conservative” take and one “Liberal” take) on 10 different political topics, and asked to select the option that best aligned with their personal beliefs. Each participant started with a score of 0. For each Conservative opinion chosen, 1 was added to their score and for each Liberal opinion chosen, −1 was added to their score, resulting in a range of scores from −10 to 10 with −10 being maximally Liberal and + 10 being maximally Conservative. We considered participants to be Liberal if their score was less than 0 and Conservative if their score was greater than 0. A potential limitation of this survey is that it restricts political opinions to those promoted in mainstream media, and excludes more radical or outside views [49]. Nevertheless, such scores capture a great deal of the variation in American political identity. Details of the demographic survey can be found in S2 and S3 Tables.

Before distributing the survey, we recieved Institutional Review Board (IRB) approval from the UC Merced IRB (IRB#: UCM2020–70). We recruited a total of 1,428 participants through Amazon Mechanical Turk. All participants had to be located in the U.S., be over 18 years old, and have a HIT Approval Rate above 95%. We inserted two check questions into our survey to gauge a user’s attentiveness to the survey in order to avoid users who randomly select choices without reading the survey content. If the individual got one or both question(s) wrong, we omitted their response. After performing omissions based upon check questions, a total of 1,244 viable participants remained. Our subsequent participant population was heavily skewed Liberal and White, while also being predominantly male. See S1 Table, S2–S6 Figs for check questions and demographic details of our participant pool.

## Results

**Fig 2:**
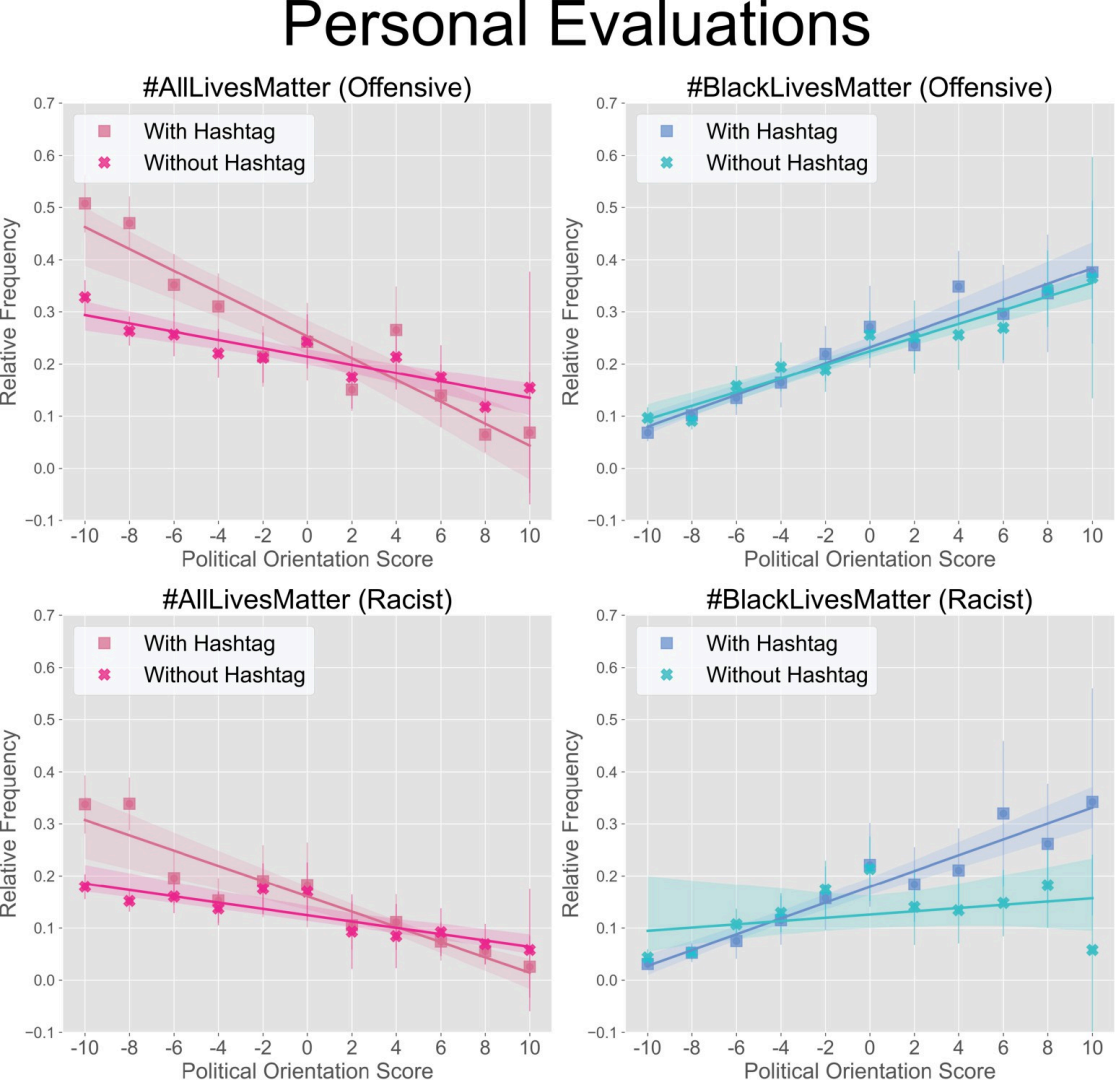
Overall personal ratings by political orientation. Relative frequencies of racist and offensive ratings for personal evaluations as a function of political score (with -10 being maximally Liberal and 10 being maximally Conservative), where relative frequency is calculated by dividing racist or offensive counts by total counts. 95% confidence is shown on relative frequencies and regressions.

To understand the relationship between demographics and corresponding evaluations, we first examined the frequency of racist and offensive ratings as a function of individuals’ demographic characteristics. Among all the demographic factors assessed, political orientation was the strongest predictor of whether tweets were perceived as racist or offensive. Perceptions of tweets marked with the #AllLivesMatter and #BlackLivesMatter hashtags were strongly mediated by political orientation, with individuals on the political left personally rating #AllLivesMatter tweets as being more offensive and racist than #BlackLivesMatter tweets. Conversely, individuals on the political right personally rated #BlackLivesMatter tweets as being more offensive and racist than #AllLivesMatter tweets. Results are shown in Fig 2.

**Fig 3:**
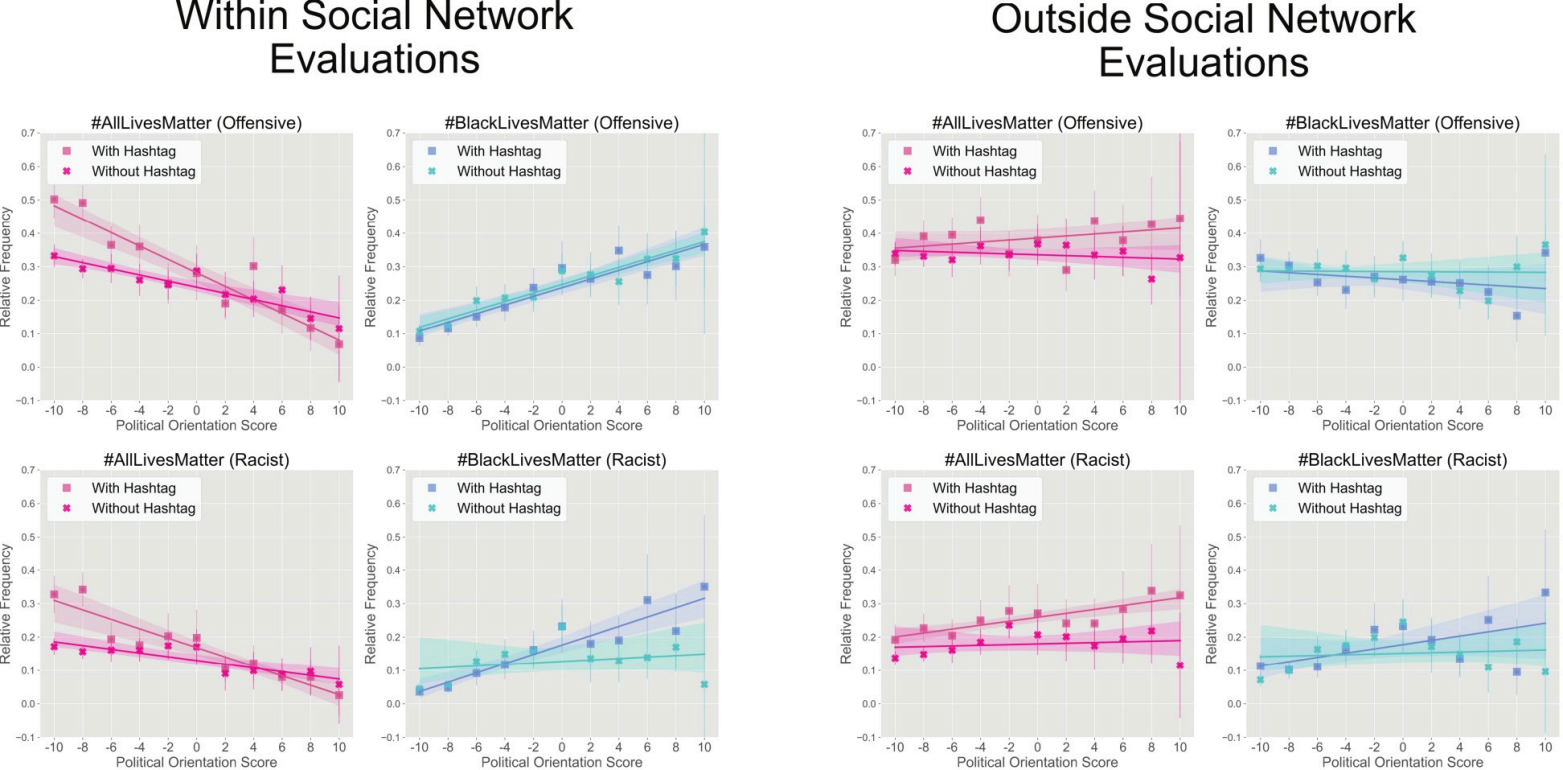
Overall “within” and “outside” personal social network ratings by political orientation. Relative frequencies of racist and offensive ratings for within personal social network and outside of personal social network evaluations as a function of political score (with -10 being maximally Liberal and 10 being maximally Conservative), where relative frequency is calculated by dividing racist or offensive counts by total counts. 95% confidence is shown on relative frequencies and regressions.

When participants were asked to imagine how individuals within their personal social networks would respond to tweets, the patterns of ratings were nearly identical to their own personal evaluations, suggesting that our participants expect cohesion and agreement with those close to them (Fig 3, left). However, the association between political orientation and perceptions of tweets as racist or offensive did not hold when participants were asked to imagine how someone outside their social network would respond, suggesting individuals understood that their judgment of the tweets as racist or offensive would not be shared by everyone (Fig 3, right).

The effect of hashtag presence was most prevalent with left leaning participants when evaluating tweets marked with #AllLivesMatter as both racist and offensive (Fig 2, left column). A similar effect was observed with right leaning participants when evaluating tweets marked with #BlackLivesMatter as racist (Fig 2, bottom right). Overall, in both cases, the presence of the hashtag made the tweet contents more likely to be perceived as racist and/or offensive by partisans. Results from independent t-tests between respective means can be found in S7 Fig.

**Fig 4:**
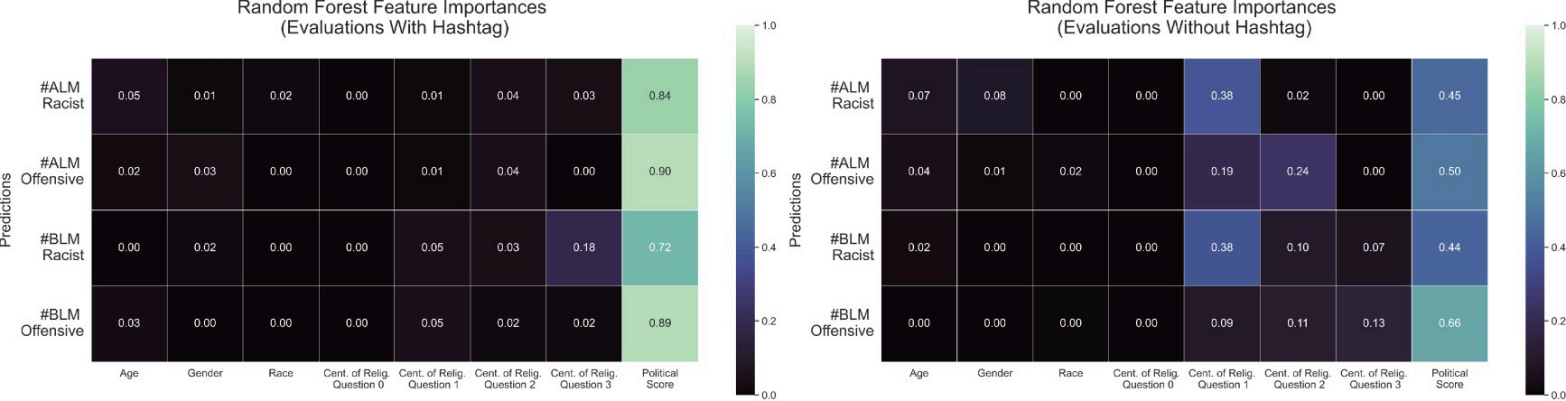
Random forest feature importances. Results of feature importances for full random forests models (using all eight predictors). Feature importances lie between [0, 1] and sum to 1, where 0 indicates a feature is not important at all and 1 indicates that a feature is as important as possible.

To verify that political orientation was the strongest predictor of how tweets were perceived, we construct two sets of models: multivariate linear regression models and random forests models to predict racist and offensive evaluations as a function of age, gender, race, four different religiosity variables, and political orientation. Full models are shown in S4 Table. For the multivariate linear regression models, we performed a partial f-test on all possible nested models (reduced models where one or more of the 8 demographic variables are removed). To evaluate the results, we examined both the f-statistic (a measure of error made by an individual nested model in terms of the residual sum of squares compared with the full model, where larger values are favorable) and p-value (a measure representing the probability that similar results would be observed if no effect was present, where smaller values are favorable). The results from the analysis can be found in S5 Table. For each partial f-test, the largest (most favorable) f-statistic corresponded to the nested models that included all variables *except* political orientation score, which shows that the nested model with the most error compared with the full model does not consider political orientation score. This indicated that political orientation score is the variable that has the strongest effect on participants’ evaluations. Corresponding p-values were very small, with a maximum of 2.1629 × 10^−6^ and a minimum of 4.7837 × 10^−27^. For random forest models, we evaluated feature importances. Results are shown in Fig 4, where each row corresponds to one model and gives the fractional amount of importance for each of the 8 feature or predictor variables, so that they sum to one. For each of the random forests models, the political orientation score is ranked substantially higher than all other predictors.

Both the multivariate linear regression models and the random forests models evince that political orientation is the strongest predictor amongst measured demographics of tweet evaluations. To analyze reliability of the tweet evaluations made by the participants, we compute intraclass correlation coefficients (ICC) [50, 51] among groups of individuals who were randomly assigned and thus evaluated the same set of tweets. The ICC is a statistical value between 0 and 1 that measures consistency of evaluations across multiple participants, with a measure of 0 indicating results are completely unreliable and a measure of 1 indicating perfect reliability. We compute ICC values from two models: a two-way random model (ICC(2, *k*)) and a two-way mixed model (ICC(3, *k*)), which differ based upon whether the groups of *k* participants are regarded as being representative of the entire population or as being the only participants of interest, respectively. In both cases, we find uniformly high values (>0.90) across datasets for both racist and offensive ratings, strongly indicating that these tweet evaluations are reliable. We report specific ICC values for racist and offensive ratings in S6 Table. Moreover, correlations between responses and other demographics (age, gender, etc.) either did not emerge in these analyses or were not significant in both of these models. We report the specific effects of various religiosity questions and participant race on resulting evaluations in S9 and S10 Figs.

**Fig 5:**
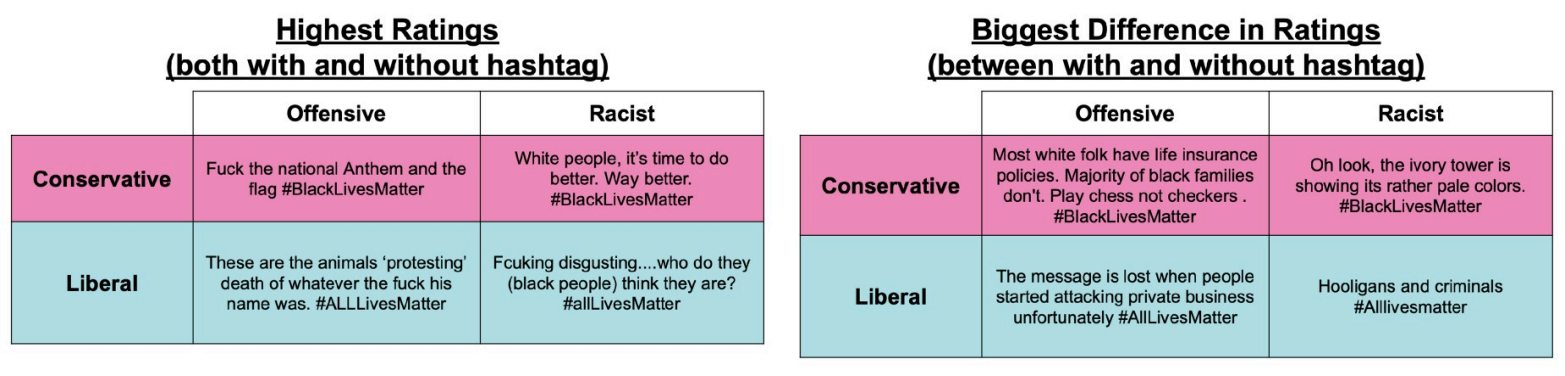
Significant tweet ratings. On left, individual tweets with the highest frequency of offensive or racist ratings, regardless of hashtag presence (relative frequencies of >0.9, >0.76, >0.86, >0.84, respectively). On right, individual tweets for which hashtag presence made the largest difference in rating frequencies (differences in relative frequencies of 0.615, 0.488, 0.426, 0.412, respectively).

The text from some of the tweets used in our study can be viewed in Fig 5. The left side of this figure shows the tweets that were consistently rated as the most offensive or racist by right and left partisans. The right side of the figure shows the tweets that exhibited the largest differences in ratings between the hashtag and no-hashtag conditions. These tweets highlight that the information content of the hashtag can vary considerably. In some cases, a hashtag simply reinforces an already-clear message, while in other cases it contextualizes and clarifies an otherwise-ambiguous message.

**Fig 6:**
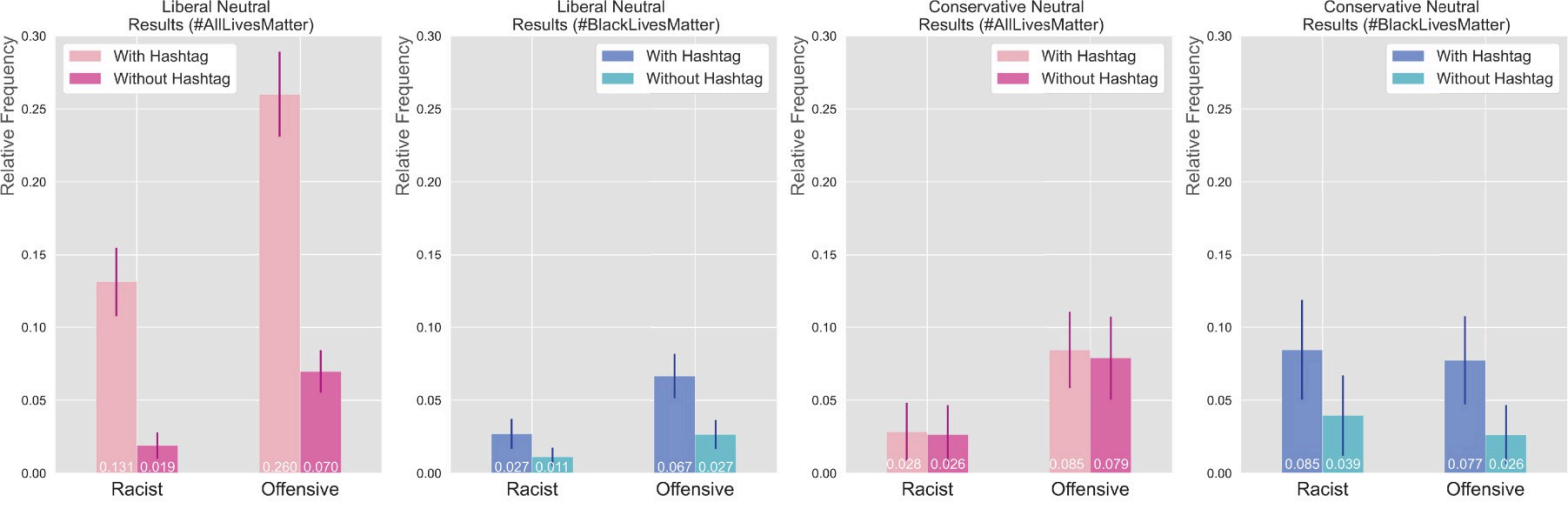
Neutral tweet evaluations. Evaluations of neutral tweets by political score with 90% confidence interval, where relative frequency is calculated by dividing racist or offensive counts by total counts. Independent t-tests revealed that there were statistically significant differences between Liberal participants evaluating neutral tweets with hashtags appended versus without, with the addition of “#AllLivesMatter” having a more significant effect (corresponding p-values of 2.359 × 10^−13^ and 1.746 × 10^−21^ for racist and offensive evaluations, respectively) than the addition of “#BlackLivesMatter” (corresponding p-values of 0.027 and 0.0002 for racist and offensive evaluations, respectively). Differences between Conservative participants evaluating neutral tweets with hashtags appended versus without were much less significant, with the addition of “#AllLivesMatter” having the weakest effect (corresponding p-values of 0.915 and 0.812 for racist and offensive evaluations, respectively), followed by the the addition of “#BlackLivesMatter” (corresponding p-values of 0.102 and 0.028 for racist and offensive evaluations, respectively).

Unsurprisingly, neutral tweets were much less likely to be rated as racist or offensive than #AllLivesMatter and #BlackLivesMatter tweets (Fig 6). However, when one of these hashtags was artificially added to a neutral tweet, that tweet was more likely to be evaluated as racist or offensive. In particular, the addition of “#AllLivesMatter” to neutral tweets was associated with a large increase in ratings as racist or offensive among Liberal participants, while the addition of “#BlackLivesMatter” to neutral tweets was associated with a moderate increase in ratings of racist and offensive among both Liberal and Conservative participants. While we found it surprising that the addition of “#BlackLivesMatter” would increase perceptions of neutral tweets as racist and offensive among Liberal participants, it is possible that such responses are provoked by the juxtaposition of something deemed quite serious (the hashtag) in a banal context.

## Discussion

In the United States and elsewhere, particularly in otherwise diverse nations, political identity is increasingly the dominant identity driving much of social behavior [19, 23, 24, 52]. Here, we have shown that among U.S. participants, perceptions of race-relevant hashtags #BlackLivesMatter and #AllLivesMatter diverge considerably in ways that are predicted by political orientation. Tweets tagged with #BlackLivesMatter were more likely to be rated as offensive and racist by participants on the political right, while tweets tagged with #AllLivesMatter were more likely to be rated as offensive and racist by participants on the political left. Political orientation was more strongly predictive of these divergent responses than any other demographic factors we examined, including the age, gender, religiosity, or race of the participants. Moreover, our results suggest that these trends are likely to be driven by identity-based assessments rather than more general perceptual differences between right and left partisans, because our main effect held when people were asked to imagine how someone else in their social networks would respond to the tweets, but not when they imagined how someone outside their social networks would response. Although other identity categories, notably historically persecuted identities associated with race and sexual orientation, are also associated with perceptions of the BLM and ALM movements in both Black and White participants [53–55], political affiliation remains the strongest predictor of that support [55].

The associations between political orientation and the tweet ratings were severely (though not entirely) diminished when the hashtags themselves were removed from the text of the tweets. However, the effect of hashtag was not consistent from tweet to tweet. In some cases, hashtags serve merely to amplify an already-clear meaning, while also increasing searchability. In other cases, however, the meaning of a tweet was ambiguous in the absence of the hashtag. In these cases, a hashtag serves to contextualize the tweet’s text and suggest a particular race-related interpretation. This role appears to have been especially important for tweets where the ratings between the hashtag and no-hashtag conditions were very different. So, although tweets marked with #BlackLivesMatter and #AllLivesMatter hashtags had stronger negative valences than neutral tweets, responses to tweets marked with these hashtags were not merely driven by the text communicated in those tweets. The hashtags themselves served as important signals, as indicated both by the diminishment of the main effect when hashtags were removed from the original tweets as well as the reintroduction of the effect when hashtags were added to neutral tweets.

Both #BlackLivesMatter and #AllLivesMatter are ostensibly about race, so it is perhaps unsurprising that removal of either hashtag reduced ratings of tweets as racist by right and left partisans, respectively. While the presence of the #BlackLivesMatter hashtags was also predictive of ratings of tweets as offensive by right partisans, these ratings appear to be driven largely by the content of the tweets themselves, and not by the hashtag. This was not the case for #AllLivesMatter, the presence of which was associated with a large increase in left partisans’ ratings of a tweet as offensive. Individuals on the political left appear to have a particularly strong reaction to the #AllLivesMatter hashtag, finding its presence offensive even when it is attached to otherwise neutral tweets. This indicates that among left partisans, #AllLivesMatter is seen not only as a marker that contextualizes other communication, but as an offensive statement in its own right. Partisans on the right may find the #BlackLivesMatter hashtag racist because they believe there is an implicit “only” in front of “black lives matter,” while left partisans may be more likely to tacitly append the statement with “too.”

The suite of views associated with political identity is not stable and particular signals are not likely to be associated with any given identity forever. Our study, however, does illuminate an association between identity, viewpoints, and signals at this point in time, which can inform our understanding of politically-relevant communication both on- and offline. More generally, our study helps to demonstrate the extent to which identity—including political identity within an allegedly integrated society—can dramatically shape how information is processed and interpreted. This can have important societal ramifications, as rational conversations about important concepts require firm grounding in how individuals are using particular terms. For example, when asked to name “socialist” countries, the top three answers given by Republican voters in the U.S. were Venezuela, China, and Russia, while the top three answers given by Democratic voters were Denmark, Sweden, and Norway [56]. Such divergent usage of the same word limits the ability of Americans to engage in meaningful dialogue about the pros and cons of socialist policies. Similarly, disagreements about what is meant by “Black Lives Matter” or “All Lives Matter”, as well as what is or is not racist or offensive is likely to hinder the ability of Americans to reach consensus or even compromise on these and related issues.

## Supporting information

S1 Fig Participant view of instructions (top) and one sample tweet evaluation (bottom) on Qualtrics. (TIF)

S2 Fig Participant responses to centrality of religiosity question 0. (TIF)

S3 Fig Participant distribution of ages with mean 39. (TIF)

S4 Fig Participant distribution of gender identities. (TIF)

S5 Fig Participant distribution of race. (TIF)

S6 Fig Distribution of political orientation scores with *μ* = 3.966. This distribution shows that the participants leaned Liberal with respect to this measure (*μ* = −3.966). Nonetheless, there are a reasonable number of participants across this political orientation spectrum to study any behavioral trends with respect to political orientation score.
(TIF)

S7 Fig P-values for independent t-tests between respective means (represented relative frequencies for with vs. without hashtag responses) within Fig 2 (in main text). (TIF)

S8 Fig Correlation between select demographics (gender, race, political score, and religiosity question results). Unsurprisingly, we found that the four religiosity scores to have the highest correlations to one another.
(TIF)

S9 Fig Evaluations of #AllLivesMatter and #BlackLivesMatter tweets by self-identification of religiosity (Centrality of religion question 0) with 95% confidence interval, where relative frequency is calculated by dividing racist or offensive counts by total counts. Independent t-tests revealed that there were statistically significant differences between evaluations of #AllLivesMatter tweets with hashtags present versus without, with the strongest effect present in evaluations of tweets as offensive (corresponding p-values of 1.514 × 10^−11^ and 0.070 for non religious and religious participant evaluations, respectively) followed by evaluations of tweets as racist (corresponding p-values of 2.605 × 10^−8^ and 0.010 for non religious and religious participant evaluations, respectively). Differences of evaluations of #BlackLivesMatter tweets with hashtags present versus without had much weaker effects, with offensive ratings (corresponding p-values of 0.442 and 0.598 for non religious and religious participant evaluations, respectively) having a slightly weaker effect than racist ratings (corresponding p-values of 0.366 and 0.164 for non religious and religious participant evaluations, respectively). These results show that religious participants tended to perceive #BlackLivesMatter tweets racist and/or offensive, particularly for tweets with hashtag present, and were less likely to find #AllLivesMatter tweets racist and/or offensive. Conversely, non-religious participants are less likely to find #BlackLivesMatter racist and/or offensive, particularly for tweets with hashtag present, and were more likely to find #AllLivesMatter racist and/or offensive. We additionally note that the presence of a hashtag has more of an effect when evaluating #AllLivesMatter tweets than #BlackLivesMatter tweets.
(TIF)

S10 Fig Evaluations of #AllLivesMatter and #BlackLivesMatter tweets by white v. non-white participants with 95% confidence interval, where relative frequency is calculated by dividing racist or offensive counts by total counts. We separated participants identifying as “white” from all others which we call “not white.” Independent t-tests revealed that there were statistically significant differences between evaluations of #AllLivesMatter tweets with hashtags present versus without, with the strongest effect present in evaluations of tweets as offensive (corresponding p-values of 8.976 × 10^−7^ and 8.967 × 10^−6^ for white and not white participant evaluations, respectively) followed by evaluations of tweets as racist (corresponding p-values of 2.456 × 10^−6^ and 0.0002 for white and not white participant evaluations, respectively). Differences of evaluations of #BlackLivesMatter tweets with hashtags present versus without had much weaker effects, with offensive ratings (corresponding p-values of 0.116 and 0.151 for non religious and religious participant evaluations, respectively) having a slightly stronger effect than racist ratings (corresponding p-values of 0.027 and 0.198 for non religious and religious participant evaluations, respectively). These results show that white participants were more likely to find #BlackLivesMatter racist and/or offensive and less likely to find #AllLivesMatter racist and/or offensive. Conversely, non-white participants were less likely to find #BlackLivesMatter racist and/or offensive and more likely to find #AllLivesMatter racist and/or offensive. The presence of hashtag has more of an effect when evaluating #AllLivesMatter tweets than #BlackLivesMatter tweets.
(TIF)

S1 Table Check questions included in the survey to gauge user attentiveness. (TIF)

S2 Table “Conservative” and “Liberal” responses used to compute political orientation scores. To measure political orientation, participants were shown two opposing opinions (one “Conservative” take and one “Liberal” take) on 10 different political topics taken from a pre-existing PEW survey [48]. Then the participants were instructed with the following: “For each of the following, select the option that aligns most with your personal beliefs”. Each participant started with a score of 0. For each Conservative opinion chosen, 1 was added to their score and for each Liberal opinion chosen, -1 was added to their score, resulting in a range of scores from −10 to 10 with -10 being as Liberal as possible and 10 being as Conservative as possible.
(TIF)

S3 Table Centrality of religiosity questions and possible responses. To measure the religiosity of each participant, we have used a subset of the Centrality of Religiosity Scale (CRS) [47], a measure of the centrality, importance or salience of religious meanings in personality.
(TIF)

S4 Table *R*^2^ values for each of the 8 full models using multivariate linear regression and random forests. (TIF)

S5 Table Results from partial f-test analysis on each of the 8 models that yield the largest f-statistics. The nested models for all of these results include all variables *except* political orientation score.
(TIF)

S6 Table Intraclass correlation coefficient (ICC) [50, 51] values for racist and offensive ratings for each dataset. Both ICC(2, *k*) (two-way random) and ICC(3, *k*) (two-way mixed) models are used.
(TIF)
