# Supplementary figures and images for: Hashtags as signals of political identity: #BlackLivesMatter and #AllLivesMatter
Source: PLoS One. 2023 Jun 8;18(6):e0286524. doi: 10.1371/journal.pone.0286524 (PMC10249887; doi:10.1371/journal.pone.0286524)

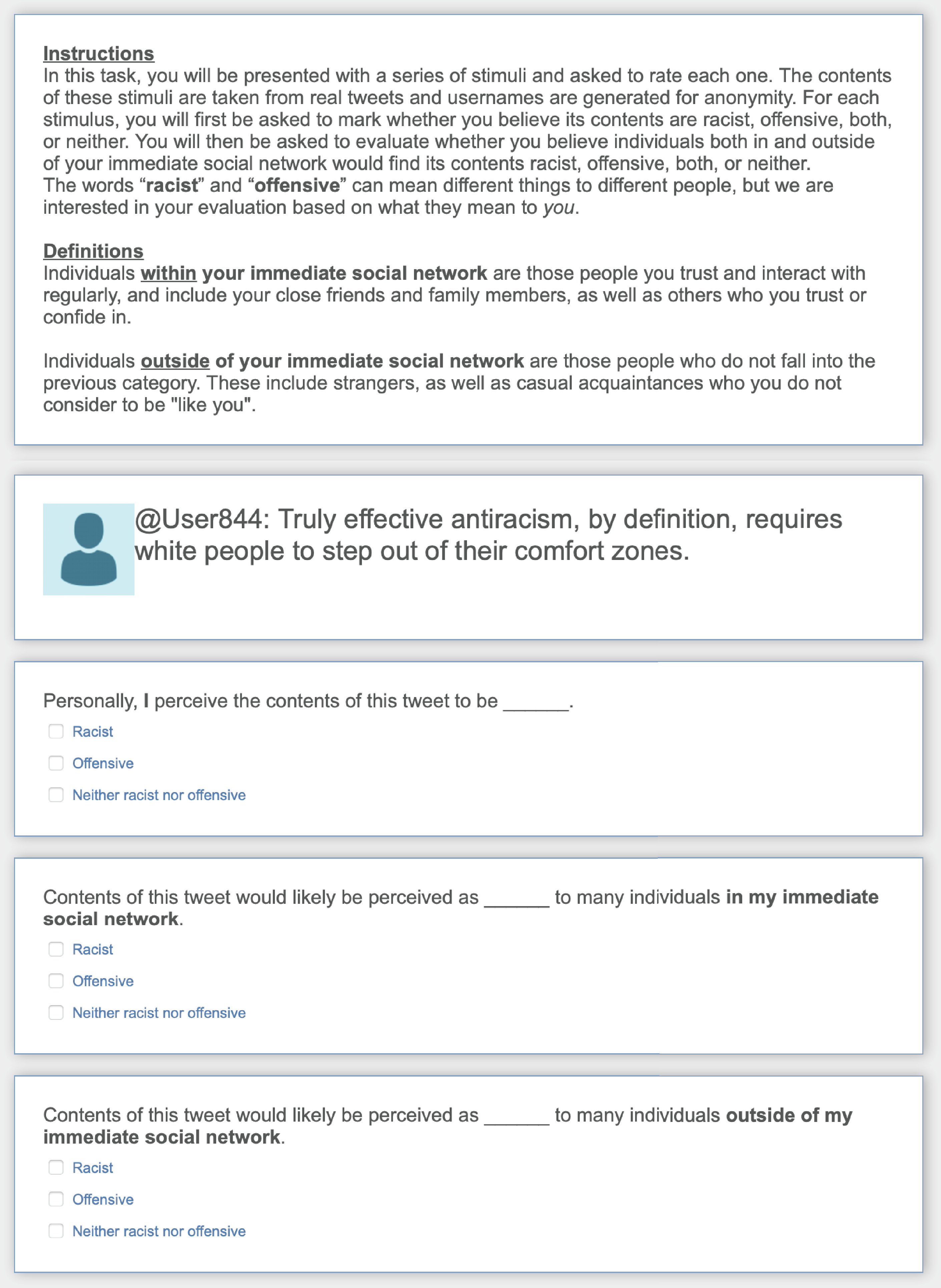

Supplement: S1 Fig — (TIF) [file pone.0286524.s001.tif]

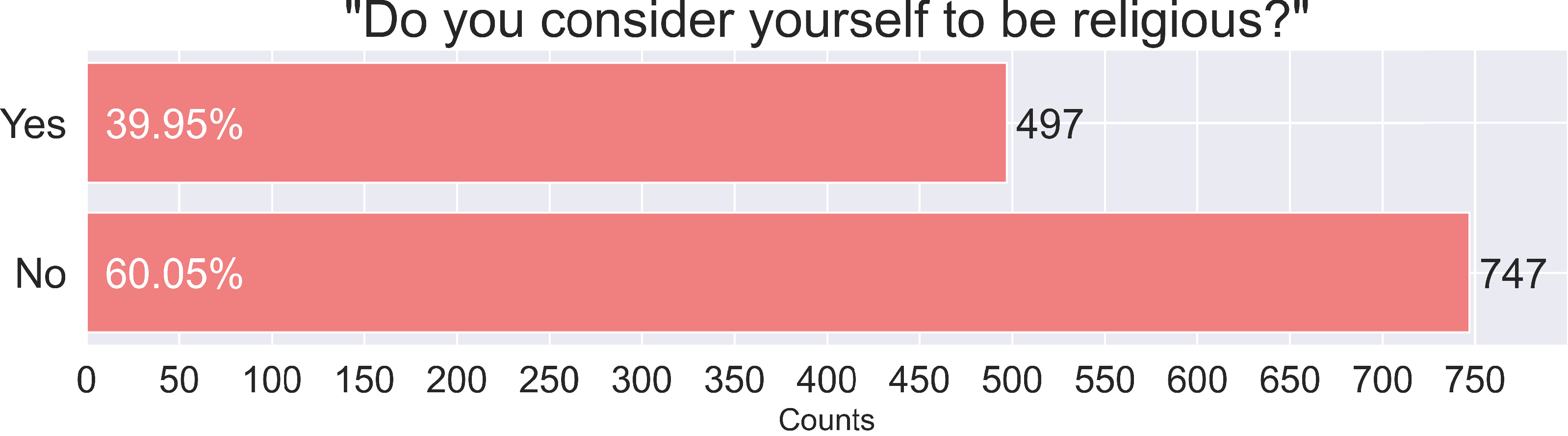

Supplement: S2 Fig — (TIF) [file pone.0286524.s002.tif]

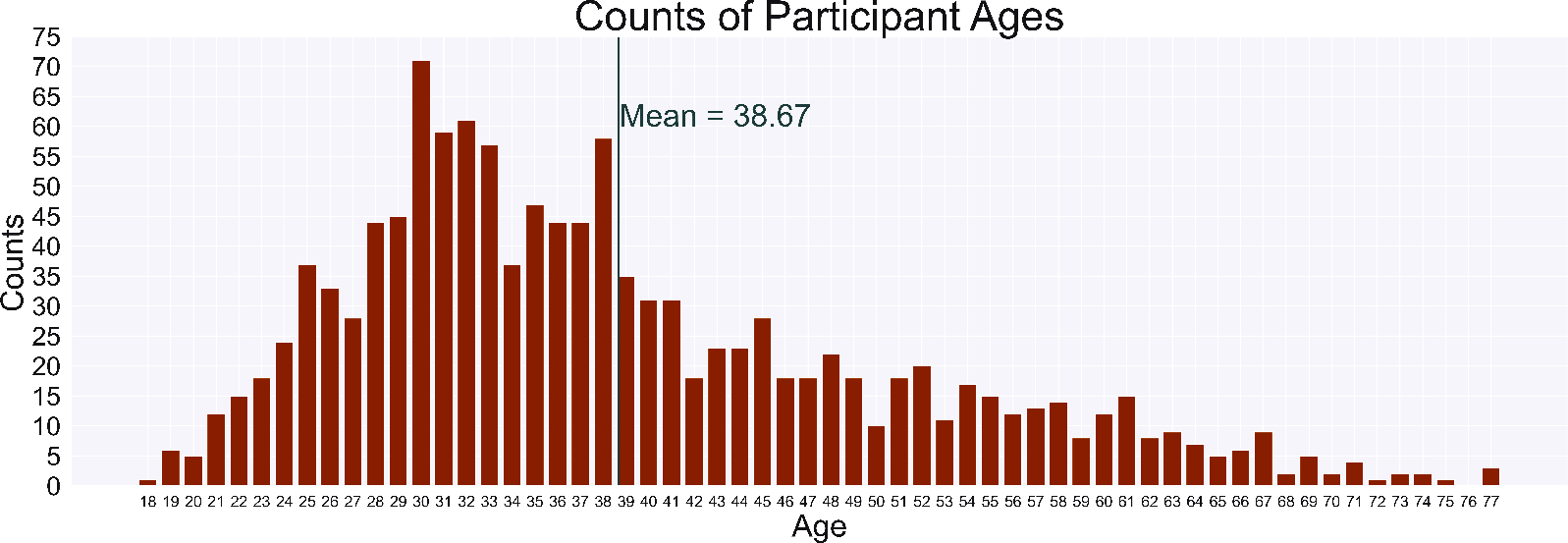

Supplement: S3 Fig — (TIF) [file pone.0286524.s003.tif]

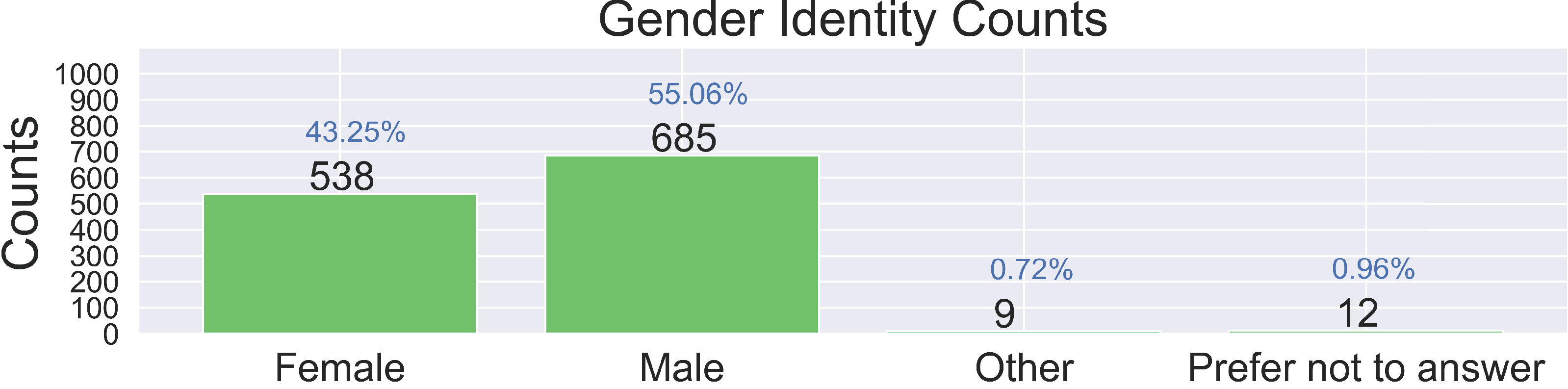

Supplement: S4 Fig — (TIF) [file pone.0286524.s004.tif]

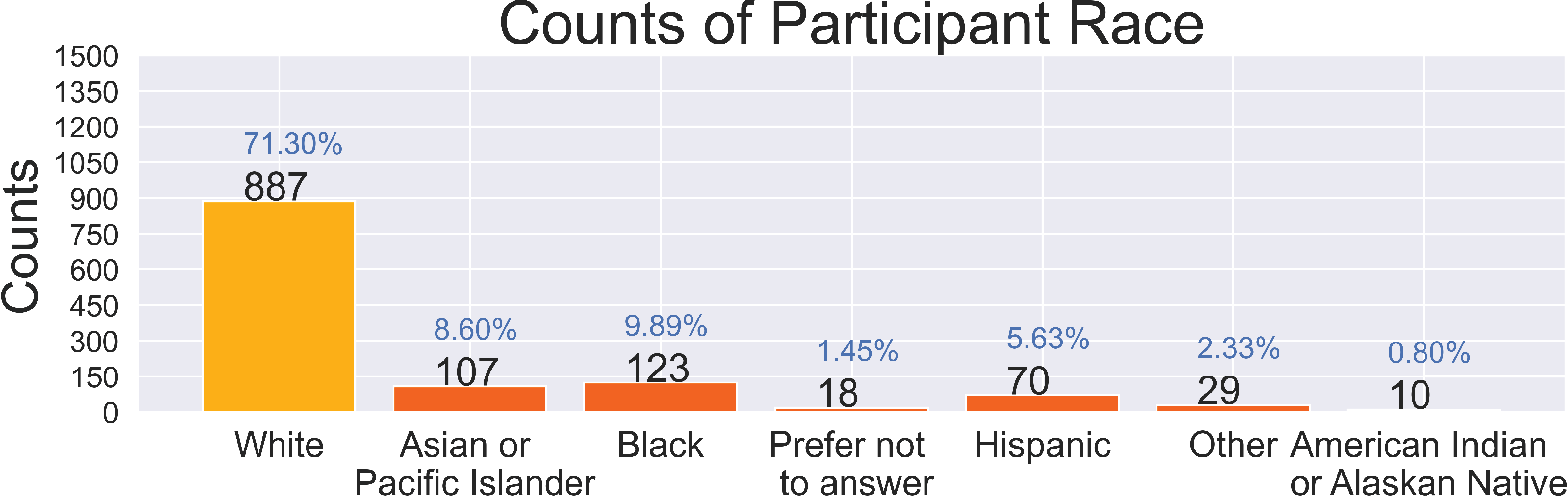

Supplement: S5 Fig — (TIF) [file pone.0286524.s005.tif]

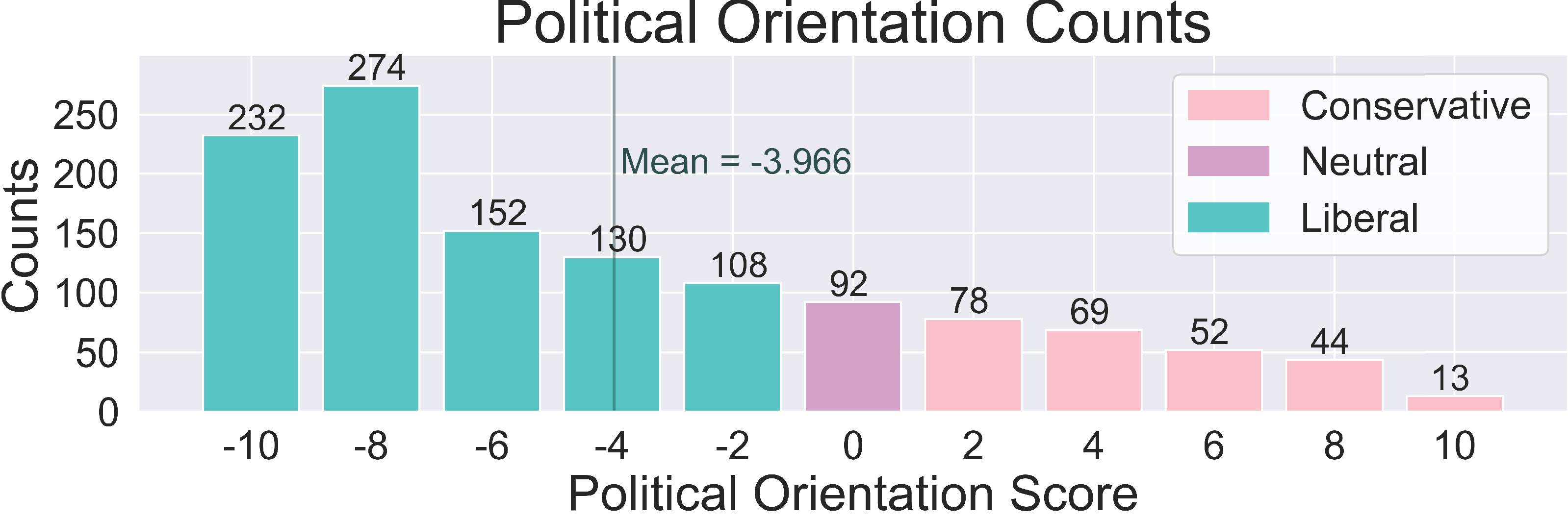

Supplement: S6 Fig — This distribution shows that the participants leaned Liberal with respect to this measure (μ = −3.966). Nonetheless, there are a reasonable number of participants across this political orientation spectrum to study any behavioral trends with respect to political orientation score. (TIF) [file pone.0286524.s006.tif]

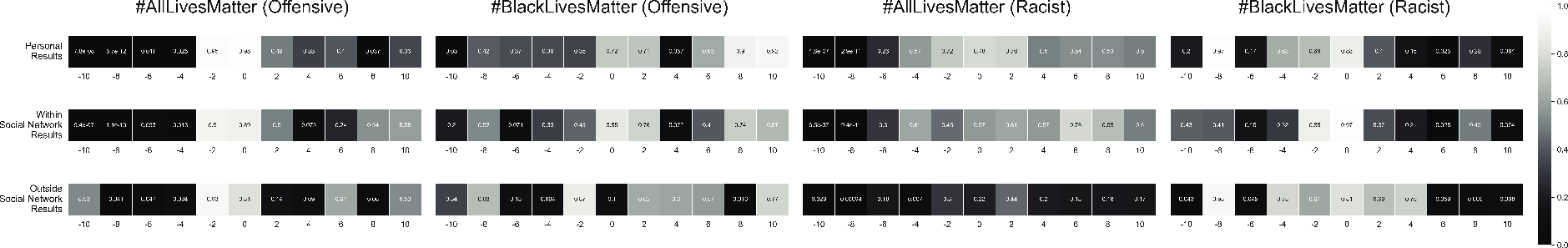

Supplement: S7 Fig — (TIF) [file pone.0286524.s007.tif]

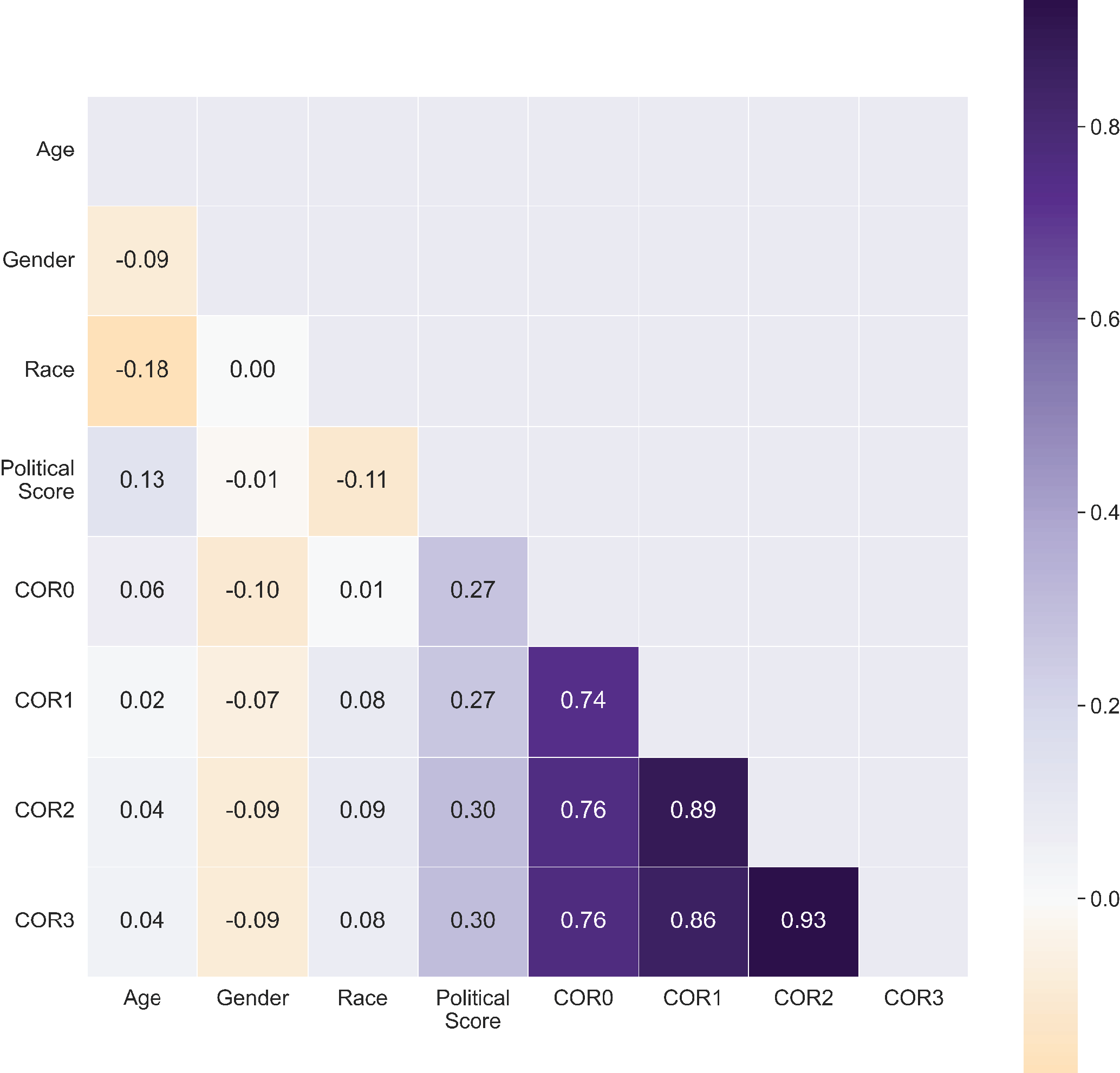

Supplement: S8 Fig — Unsurprisingly, we found that the four religiosity scores to have the highest correlations to one another. (TIF) [file pone.0286524.s008.tif]

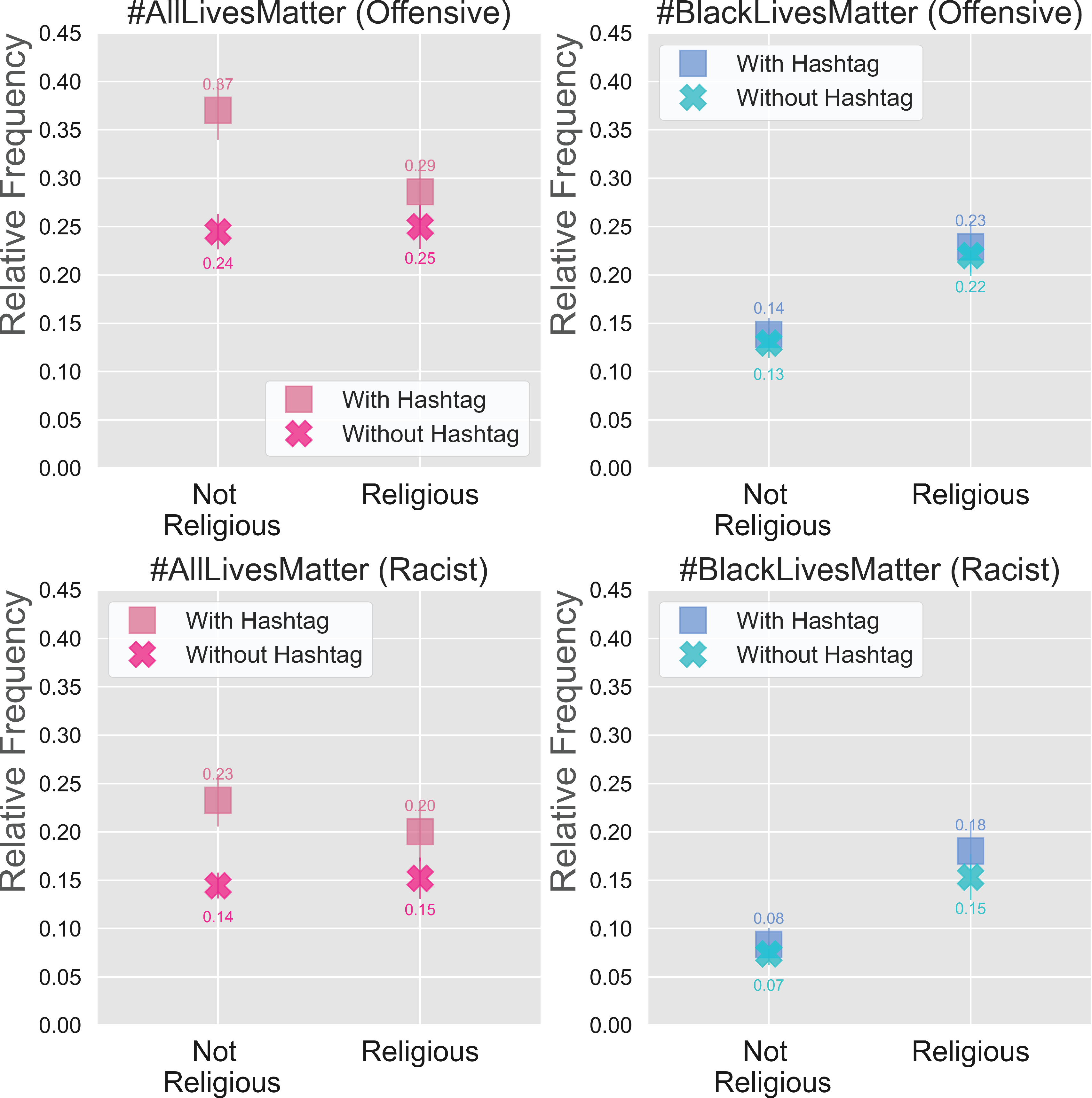

Supplement: S9 Fig — Independent t-tests revealed that there were statistically significant differences between evaluations of #AllLivesMatter tweets with hashtags present versus without, with the strongest effect present in evaluations of tweets as offensive (corresponding p-values of 1.514 × 10^−11^ and 0.070 for non religious and religious participant evaluations, respectively) followed by evaluations of tweets as racist (corresponding p-values of 2.605 × 10^−8^ and 0.010 for non religious and religious participant evaluations, respectively). Differences of evaluations of #BlackLivesMatter tweets with hashtags present versus without had much weaker effects, with offensive ratings (corresponding p-values of 0.442 and 0.598 for non religious and religious participant evaluations, respectively) having a slightly weaker effect than racist ratings (corresponding p-values of 0.366 and 0.164 for non religious and religious participant evaluations, respectively). These results show that religious participants tended to perceive #BlackLivesMatter tweets racist and/or offensive, particularly for tweets with hashtag present, and were less likely to find #AllLivesMatter tweets racist and/or offensive. Conversely, non-religious participants are less likely to find #BlackLivesMatter racist and/or offensive, particularly for tweets with hashtag present, and were more likely to find #AllLivesMatter racist and/or offensive. We additionally note that the presence of a hashtag has more of an effect when evaluating #AllLivesMatter tweets than #BlackLivesMatter tweets. (TIF) [file pone.0286524.s009.tif]

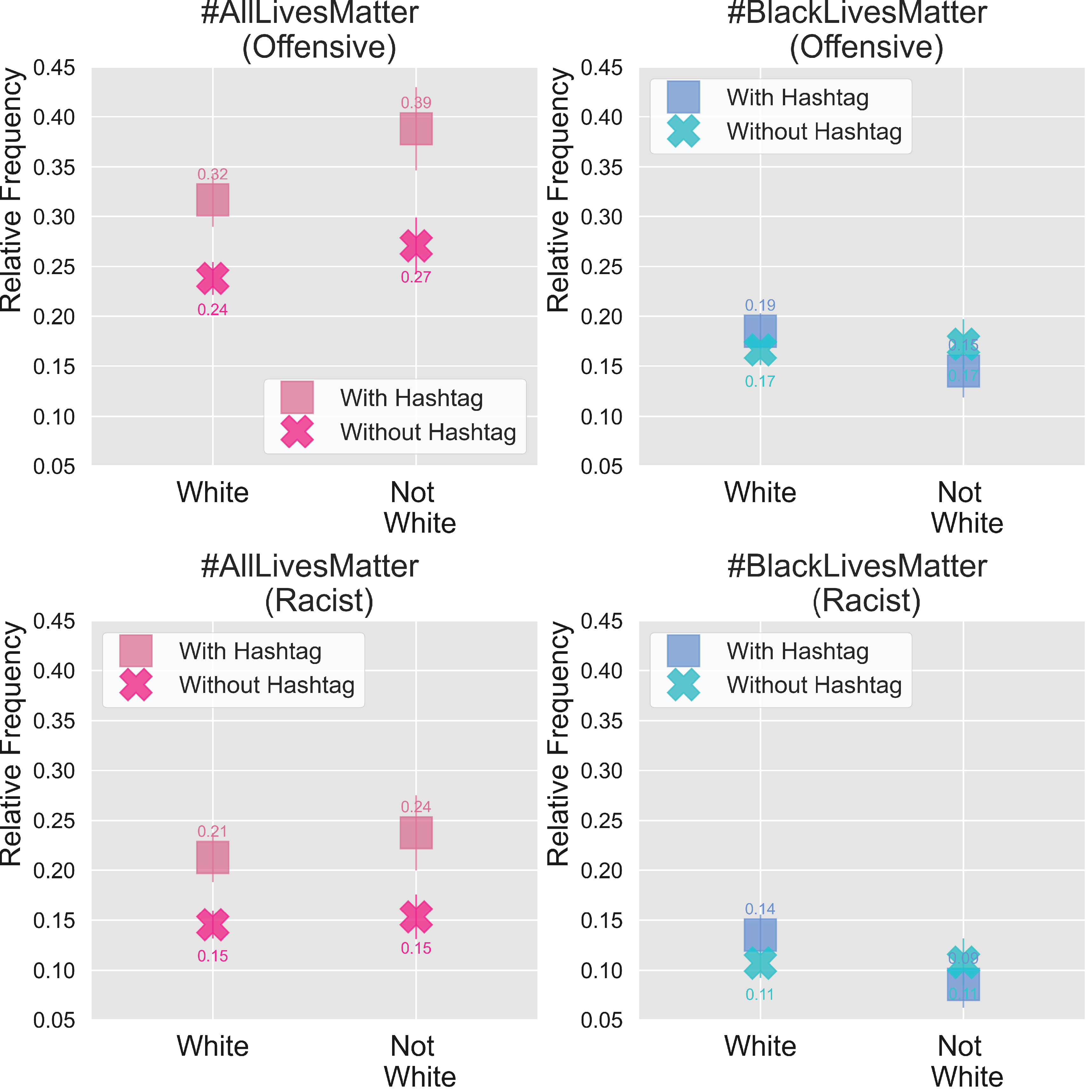

Supplement: S10 Fig — We separated participants identifying as “white” from all others which we call “not white.” Independent t-tests revealed that there were statistically significant differences between evaluations of #AllLivesMatter tweets with hashtags present versus without, with the strongest effect present in evaluations of tweets as offensive (corresponding p-values of 8.976 × 10^−7^ and 8.967 × 10^−6^ for white and not white participant evaluations, respectively) followed by evaluations of tweets as racist (corresponding p-values of 2.456 × 10^−6^ and 0.0002 for white and not white participant evaluations, respectively). Differences of evaluations of #BlackLivesMatter tweets with hashtags present versus without had much weaker effects, with offensive ratings (corresponding p-values of 0.116 and 0.151 for non religious and religious participant evaluations, respectively) having a slightly stronger effect than racist ratings (corresponding p-values of 0.027 and 0.198 for non religious and religious participant evaluations, respectively). These results show that white participants were more likely to find #BlackLivesMatter racist and/or offensive and less likely to find #AllLivesMatter racist and/or offensive. Conversely, non-white participants were less likely to find #BlackLivesMatter racist and/or offensive and more likely to find #AllLivesMatter racist and/or offensive. The presence of hashtag has more of an effect when evaluating #AllLivesMatter tweets than #BlackLivesMatter tweets. (TIF) [file pone.0286524.s010.tif]

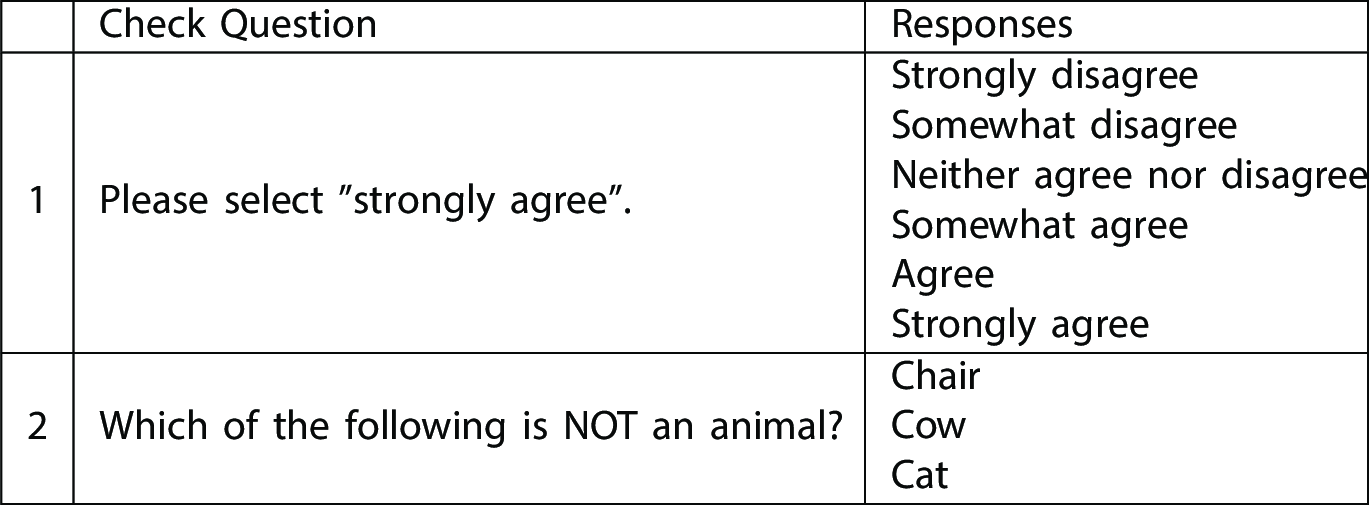

Supplement: S1 Table — (TIF) [file pone.0286524.s011.tif]

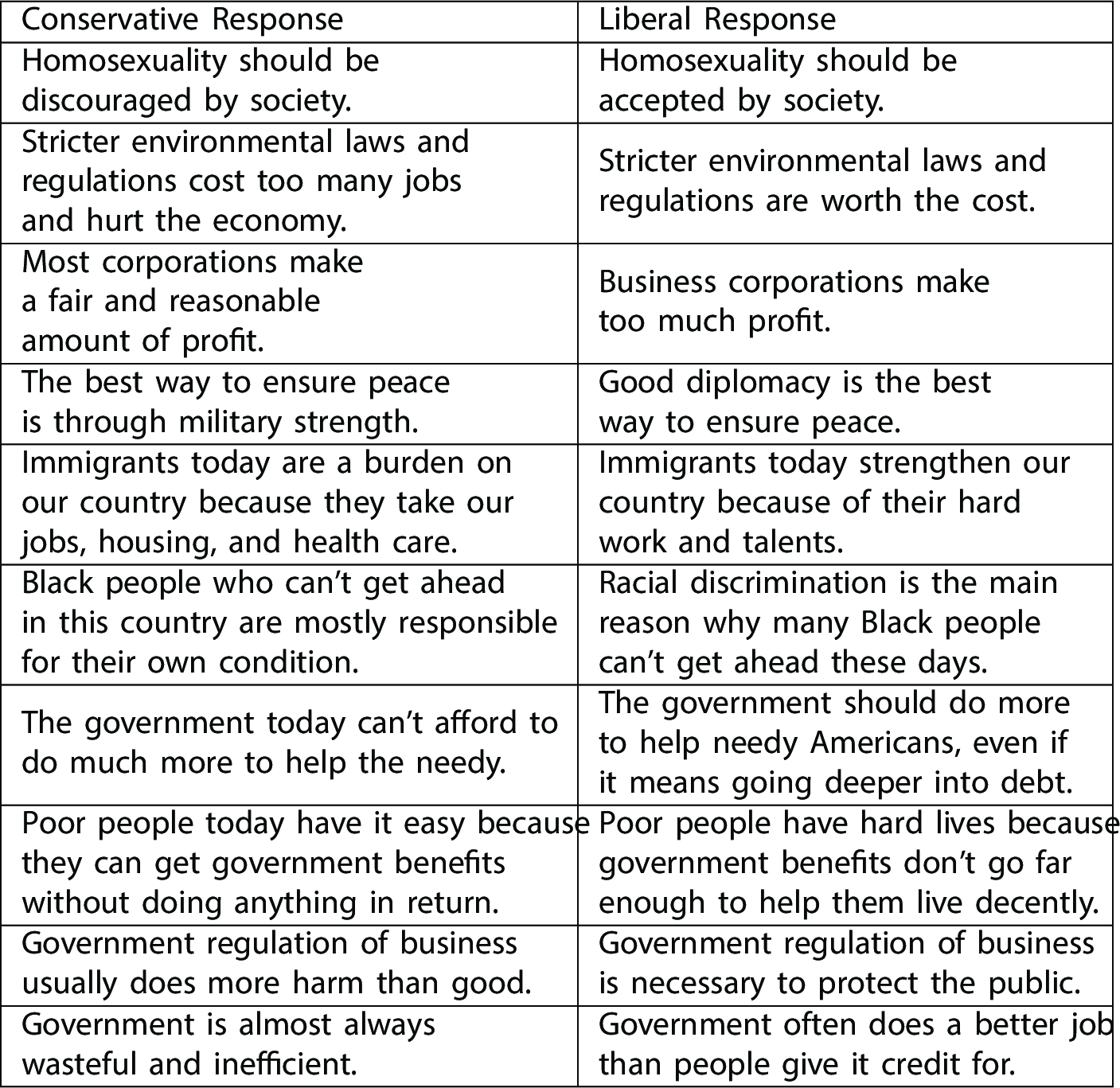

Supplement: S2 Table — To measure political orientation, participants were shown two opposing opinions (one “Conservative” take and one “Liberal” take) on 10 different political topics taken from a pre-existing PEW survey [48]. Then the participants were instructed with the following: “For each of the following, select the option that aligns most with your personal beliefs”. Each participant started with a score of 0. For each Conservative opinion chosen, 1 was added to their score and for each Liberal opinion chosen, -1 was added to their score, resulting in a range of scores from −10 to 10 with -10 being as Liberal as possible and 10 being as Conservative as possible. (TIF) [file pone.0286524.s012.tif]

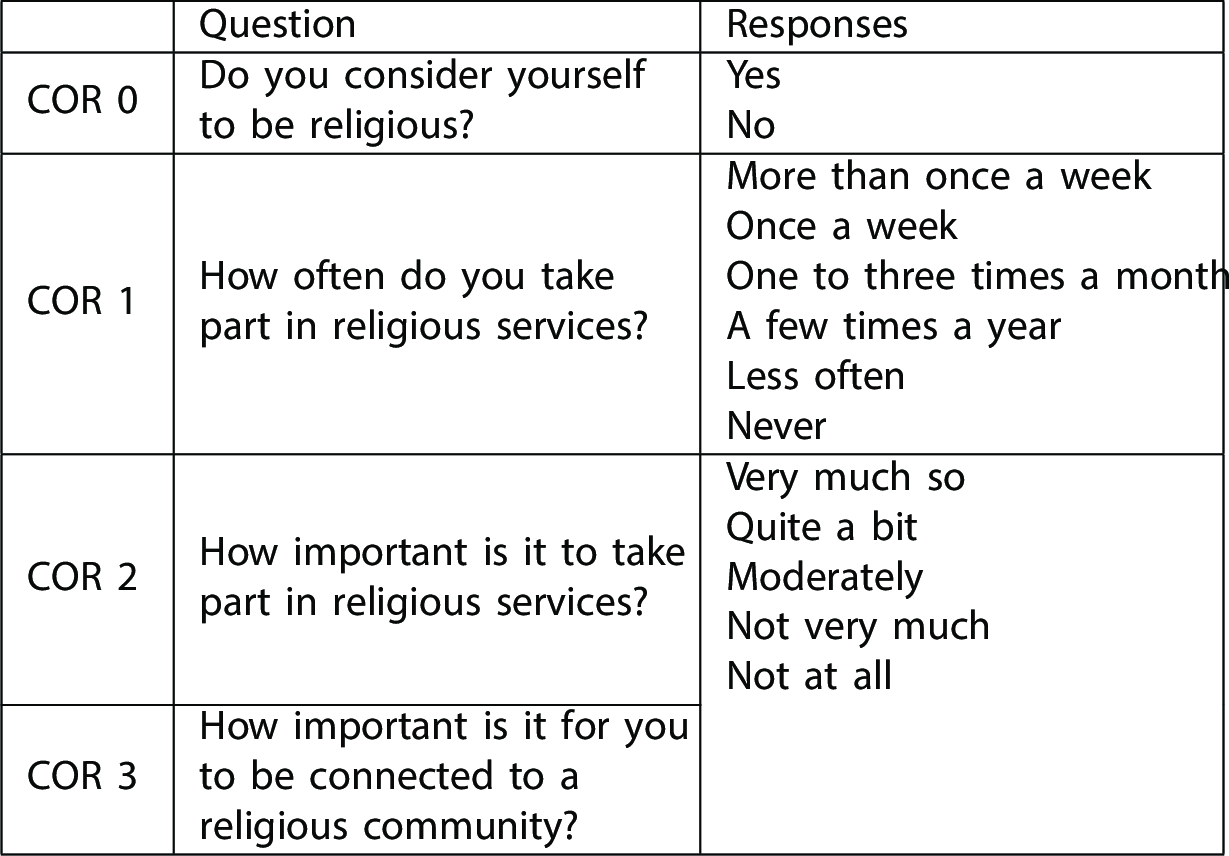

Supplement: S3 Table — To measure the religiosity of each participant, we have used a subset of the Centrality of Religiosity Scale (CRS) [47], a measure of the centrality, importance or salience of religious meanings in personality. (TIF) [file pone.0286524.s013.tif]

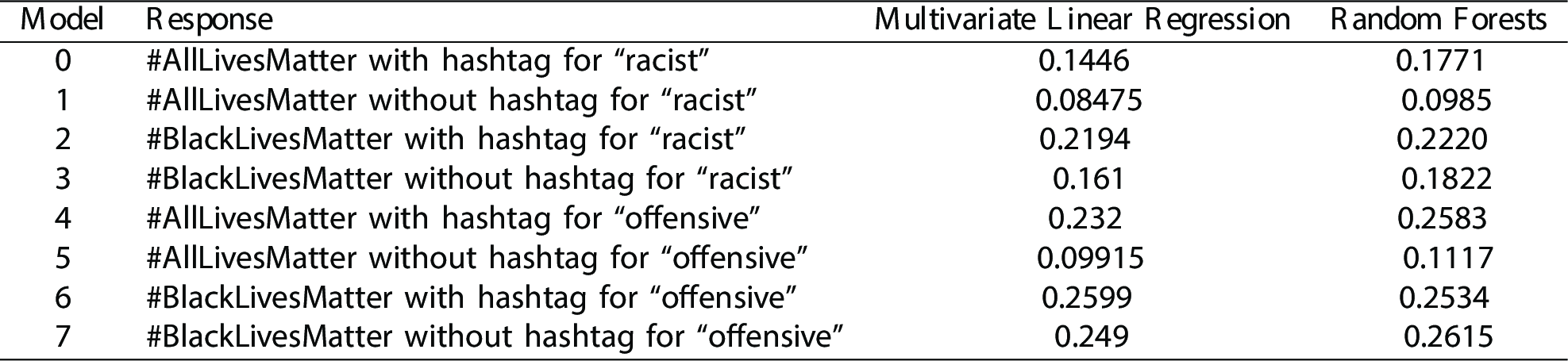

Supplement: S4 Table — (TIF) [file pone.0286524.s014.tif]

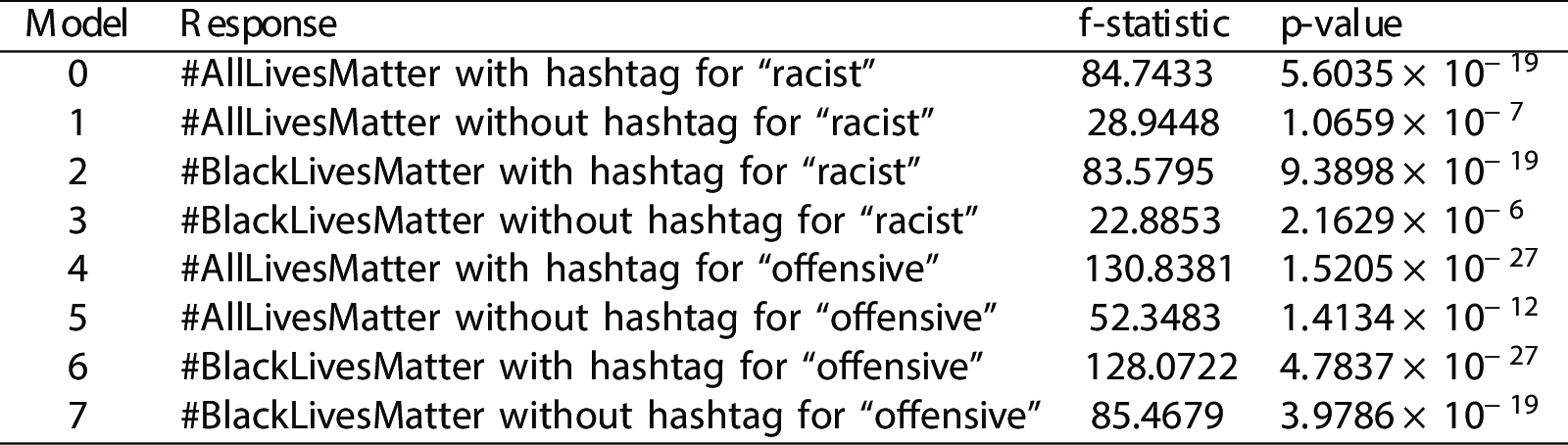

Supplement: S5 Table — The nested models for all of these results include all variables except political orientation score. (TIF) [file pone.0286524.s015.tif]

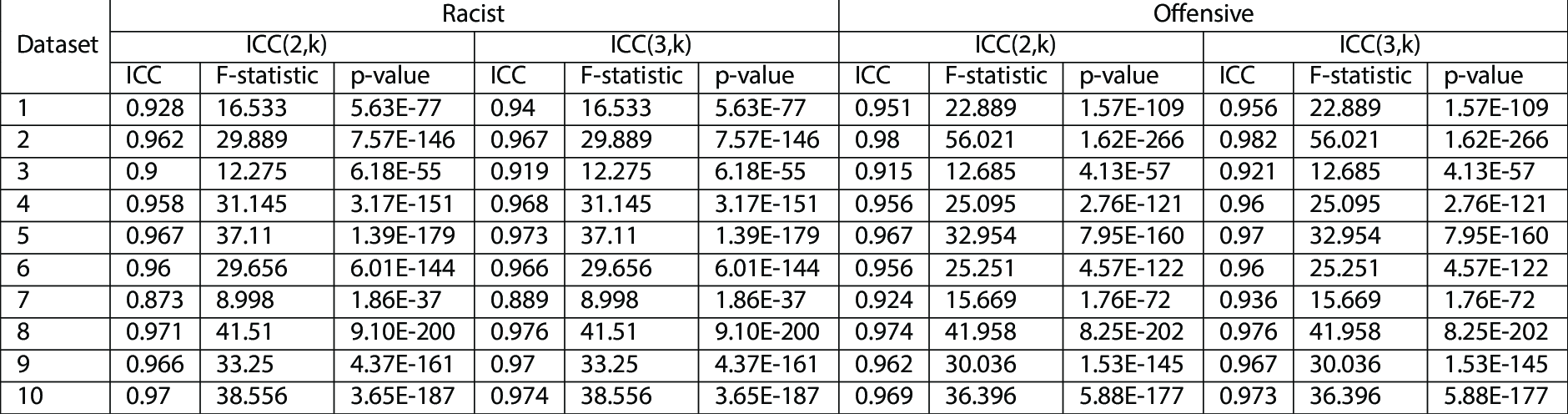

Supplement: S6 Table — Both ICC(2, k) (two-way random) and ICC(3, k) (two-way mixed) models are used. (TIF) [file pone.0286524.s016.tif]
